# Scaling the consequences of interactions between invaders from the individual to the population level

**DOI:** 10.1002/ece3.2008

**Published:** 2016-02-18

**Authors:** Blaine D. Griffen

**Affiliations:** ^1^ Department of Biological SciencesUniversity of South CarolinaColumbiaSouth Carolina29208; ^2^ Marine Science ProgramUniversity of South CarolinaColumbiaSouth Carolina29208

**Keywords:** Anthropogenic stressors, *Carcinus maenas*, fecundity, invasive species, life table, predation

## Abstract

The impact of human‐induced stressors, such as invasive species, is often measured at the organismal level, but is much less commonly scaled up to the population level. Interactions with invasive species represent an increasingly common source of stressor in many habitats. However, due to the increasing abundance of invasive species around the globe, invasive species now commonly cause stresses not only for native species in invaded areas, but also for other invasive species. I examine the European green crab *Carcinus maenas*, an invasive species along the northeast coast of North America, which is known to be negatively impacted in this invaded region by interactions with the invasive Asian shore crab *Hemigrapsus sanguineus*. Asian shore crabs are known to negatively impact green crabs via two mechanisms: by directly preying on green crab juveniles and by indirectly reducing green crab fecundity via interference (and potentially exploitative) competition that alters green crab diets. I used life‐table analyses to scale these two mechanistic stressors up to the population level in order to examine their relative impacts on green crab populations. I demonstrate that lost fecundity has larger impacts on per capita population growth rates, but that both predation and lost fecundity are capable of reducing population growth sufficiently to produce the declines in green crab populations that have been observed in areas where these two species overlap. By scaling up the impacts of one invader on a second invader, I have demonstrated that multiple documented interactions between these species are capable of having population‐level impacts and that both may be contributing to the decline of European green crabs in their invaded range on the east coast of North America.

## Introduction

Natural systems today experience a wide range of human‐induced rapid environmental changes brought about by stressors such as pollution, habitat destruction, exploitation, and climate change. For individual organisms, these stressors can have large detrimental impacts on physiological condition (e.g., Bayne et al. 1979), growth (e.g., Dobbertin 2005), fecundity (e.g., Schreck et al. 2001), or survival (e.g., Pickering 1989). Measuring and mechanistically understanding these impacts of environmental stressors on individual organisms is relatively straightforward. However, for management or broader scale ecological understanding, it is often desirable to scale up from these individual‐level responses to population‐level impacts. And while the response of individual organisms to these stressors can be easily studied and quantified, population‐level responses to many stressors are largely unknown (Walker et al. 2012).

One increasingly common type of stressor is the introduction of invasive species in habitats around the globe. Invasive species can have large detrimental impacts on individual organisms in invaded areas that are well documented. For instance, as with negative impacts of environmental stressors more broadly, interactions with invasive species can lead to impaired physiological condition (e.g., Narayan et al. 2015), reduced growth (e.g., Pearson et al. 2015), lower fecundity (e.g., Miller and Gorchov 2004; Narayan et al. 2015), and decreased survival (e.g., Albins 2015) for individual organisms in invaded areas. These negative impacts can alter the evolutionary trajectory of impacted populations (Mooney and Cleland 2001), for example, by altering the success of existing life‐history strategies (e.g., Griffen and Riley 2015). Together, these negative impacts of invasive species can even be drivers of extinction for native species (Gurevitch and Padilla 2004), although this conclusion has been questioned (Ricciardi 2004; Clavero and García‐Berthou 2005).

With the ubiquity of invaders and their continued introductions around the world, invaders are commonly interacting not just with native species, but with each other as multiple invaders get introduced into the same region. While invaders may potentially facilitate each other's success (i.e., the invasional meltdown hypothesis), existing evidence indicates that it is much more common for at least one species in a pair of interacting invaders to be negatively influenced by the interaction (Simberloff and Von Holle 1999). However, while the impacts of invasive species, both on native species and on other invasive species, are commonly documented at the individual level, scaling these individual‐level impacts up to the population level is much less common. A case in point is the interaction between two species of invasive crabs in intertidal regions along the New England coast of North America.

The European green crab *Carcinus maenas* became established along the northeast coast of North America beginning in the early 1800s (Say 1817) where it thrived and continued to spread for two centuries (Audet et al. 2008). In 1989, the Asian shore crab *Hemigrapsus sanguineus* was introduced to the same region (McDermott 1991) and the preferred habitats of these two species overlap extensively in rocky intertidal areas. The Asian shore crab has generally had the upper hand in interactions between these species. For instance, the Asian crab is known to directly consume young green crab juveniles (Lohrer and Whitlatch 2002), displace the green crab from refuge habitat (Jensen et al. 2002), steal food from the green crab (Jensen et al. 2002), and cause the green crab to alter its foraging strategy in ways that reduce its overall food intake and to replace a diet composed primarily of animal tissue with one composed primarily of plant material (Griffen et al. 2008). These changes in food consumption lead to a subsequent decline in reproductive success for individual green crabs (Griffen et al. 2011). Ultimately, these negative impacts have led to a decline in green crab abundance and to their nearly complete disappearance from rocky shores where these two species interact (Lohrer and Whitlatch 2002; Kraemer et al. 2007). However, interactions with the Asian shore crab represent multiple mechanisms of stress for green crabs: predation threat and reduced reproductive success; and it is unclear how these two mechanisms scale up to the population level.

Scaling the impacts of predation and reduced fecundity to the population level requires a robust understanding of each of these impacts. In terms of predation, the impacts at the individual level are fairly straight forward. The greatest predation risk for juvenile green crabs is generally from cannibalism (Moksnes et al. 1998). Cannibalism is strongest on newly settled 0‐year class juveniles, and drops off quickly as individuals grow and become more robust and capable of fleeing or defending themselves (Moksnes et al. 1998). Additionally, the risk of cannibalism changes with crab density and with habitat structure and is therefore condition dependent (Moksnes 2004). Previous field experiments demonstrate that predation by Asian shore crabs on green crabs also differs with crab size, with habitat complexity and with other conditions (Lohrer and Whitlatch 2002). However, these experiments show that across all conditions examined, predation from Asian shore crabs produces mortality rates for green crabs that are nearly identical to mortality from cannibalism (Lohrer and Whitlatch 2002).

In terms of reduced fecundity from altered diets, our understanding is not as complete. The contribution of animal tissue to the diet generally increases with green crab size (Ropes 1968). Additionally, food consumption required to meet metabolic demands sharply increases with crab size (Elner and Hughes 1978), and reproductive output increases linearly with the amount of animal tissue consumed, but is not influenced appreciably by algal consumption (Griffen 2014). Previous field experiments demonstrate that interactions with Asian shore crabs cause green crabs to reduce their food consumption overall, with this reduction reflecting primarily a loss of animal tissue consumption (Griffen et al. 2008). However, it is possible that reproductive implications of these dietary changes vary with the size of green crabs, given the increasing nutritional/energetic needs of crabs with size (Elner and Hughes 1978) and the general increased reliance on animal tissue for larger individuals (Ropes 1968). Efforts to scale Asian shore crab impacts on individual green crab fecundity to the population level may therefore benefit from knowing whether the contribution of animal consumption still scales with green crab body size, even in the presence of Asian shore crabs.

Finally, fecundity of crabs generally increases with size due to allometric constraints of space for ovary development inside the carapace (Hines 1982). Green crabs also follow this pattern and clutch size has been shown to vary from 140,000 to 200,000 eggs, depending of the size of the female (Audet et al. 2008), with an average‐sized female of 46‐mm carapace width producing 185,000 eggs (Broekhuysen 1936). Efforts to scale Asian shore crab impacts on individual green crab fecundity to the population level may therefore also benefit from knowing the size–fecundity relationship for green crabs in the presence of Asian shore crabs.

I examined how the relative amount of animal consumption by adult female green crabs varies with crab size at two sites on the New Hampshire coast where these two species overlap. I also examined the size–fecundity relationship for green crabs at these same sites. Finally, I used a life‐table analysis to examine the relative population‐level impacts of predation by Asian shore crabs on 0‐year class green crabs and of reduced fecundity of green crabs in the presence of Asian shore crabs.

## Methods

### Field population sampling

I sampled crabs from two sites on the New Hampshire coast (Fort Stark and Odiorne Point State Park) that are separated by approx. 2.5 km. See Tyrrell and Harris (2000) for a general description of these two sites. While there are physical and biological differences between these two sites, there were no differences between these sites in the relationships examined here (i.e., size vs. animal consumption, and size vs. fecundity). For simplicity of presentation, I therefore pool data from these two sites in all descriptions and analyses below. The Asian shore crab is now abundant at both of these sites. I sampled each of these sites by exhaustively searching each for 12 h spread over 2 days during low tide (Fort Stark: 27, 29 April 2009; Odiorne Point: 28, 30 April 2009). I collected all mature‐sized (≥29 mm carapace width, CW) female green crabs encountered. Upon collection, crabs were immediately frozen. They were then returned to the University of South Carolina for complete analysis.

### Size versus diet

I assessed individual long‐term diet variation using stable nitrogen isotope values that have previously been used to examine the correlation between relative trophic position and reproductive success (Griffen 2014). Here, I use these same data to examine the relationship between crab size (carapace width, CW) and relative trophic position, which has not previously been explored. *δ* ^15^N values generally increase with trophic position and can therefore accurately be used to assess relative trophic position between individuals (Post 2002). *δ* ^15^N values were obtained from muscle tissue taken from a single walking leg of each crab and were measured using an Isoprime mass spectrometer connected via continuous flow to a EuroVector Elemental Analyzer. Three internal standards were run approximately every 40 samples to calibrate the system and to compensate for potential drift over time (USGS40, N1 and N2). I tested the hypothesis that relative trophic position would increase with crab size using a linear model with *δ* ^15^N as the response variable and crab CW as predictor variable.

### Size versus fecundity

**Figure 1:**
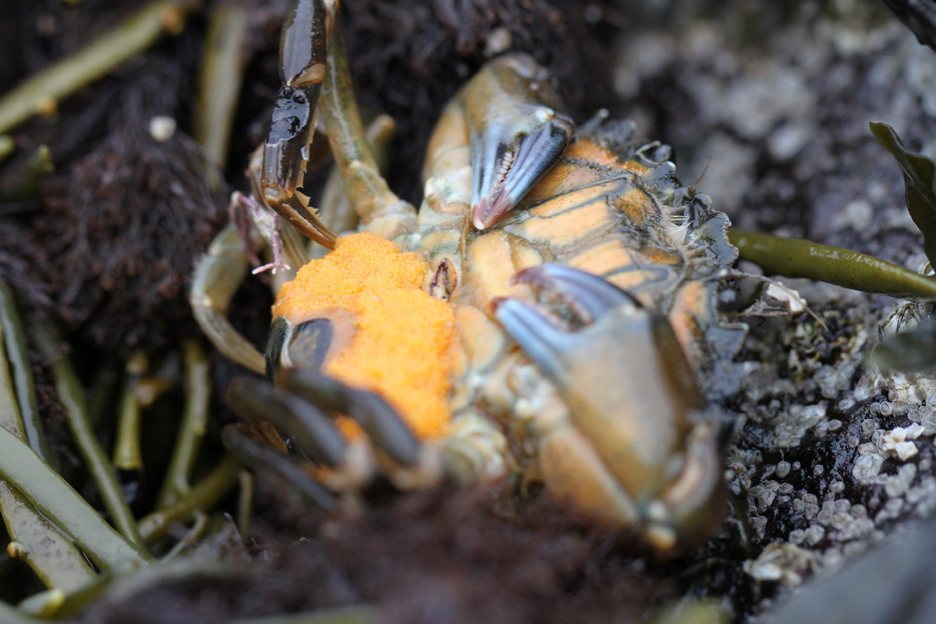
Gravid female European green crab *Carcinus maenas*.

To facilitate comparison with size–fecundity relationships previously reported for this species (Audet et al. 2008), I examined clutch size of green crabs as a function of the abdomen width (AW), measured at the base of the abdomen at its widest point. I determined clutch size by first determining the dry weight of the egg mass from each gravid crab after drying at 70°C for 72 h. I then divided the clutch mass by the mass of a single egg (0.00001373 g, Griffen 2014) to yield the total number of eggs present. I then determined the size–fecundity relationship using a linear model with number of eggs treated as the response variable and abdomen width as the predictor variable (Fig. 1).

For comparison, I also calculated the predicted number of eggs for each crab based on its size using the following relationship given by Audet et al. (2008) (*R* ^2^ = 0.916) for green crabs that occur further north in the absence of Asian shore crabs:

(1) Number eggs=25531×AW−347,866

I then compared this predicted number of eggs to the number that I observed using a paired *t*‐test.

### Scaling from individual to population impacts

I used a life‐table analysis to examine the relative impacts of predation mortality and loss of fecundity on green crab populations that overlap with Asian shore crabs. I first established a life table for green crabs from historic data at this site using data collected by Tyrrell (2002) for the 5 years immediately preceding the arrival of the Asian shore crab at Odiorne Point, NH (Table 1a). Values for this life table were derived from previously documented life‐history characteristics for this species. Specifically, this life table was based on the finding that green crabs become sexually mature in their third year in this region (Berrill 1982) and that the maximum size of immature year 1 and year 2 green crabs are, respectively, 10 and 28 mm (Berrill 1982). I then calculated the maximum size of year 3, year 4, and year 5 crabs based on the finding that green crabs molt annually once sexual maturity is reached (Broekhuysen 1936) and that they grow by 29% with each molt (Crothers 1967). To calculate fecundity, I converted the maximum CW of each year class to abdomen width (AW) using the following equation derived from Fig. 13A of Audet et al. (2008) (*r* ^2^ > 0.95):

(2) AW=0.38×CW+1.6487.

**Table 1:** Life tables used to examine the impacts of the Asian shore crab on green crab populations. Part (a) gives the standard life table based on historical data from the mean of 5 years of sampling prior to the invasion of Asian shore crabs to Odiorne State Park, NH, from Tyrrell (2002). Parts (b–d) show the equations for the calculated life tables that include the impacts of predation by Asian shore crabs on 0‐year class green crabs (Part (b)), reduced green crab fecundity following interactions with Asian shore crabs (Part (c)), or both of these impacts simultaneously (Part (d)). See text for explanation and for further equations

Age/Stage	(a) Historic			(b) Predation		
	*a*_*x,H*_	*l*_*x,H*_	*m*_*x,H*_	*a*_*x,P*_	*l*_*x,P*_	*m*_*x,P*_
Eggs	55,822	—	—	*E*_*H*_	—	—
0 year	32.168	5.763E‐4	—	*a*_*0,H*_	*a*_*0,P*_/*E*_*H*_	—
1 year	5.806	1.040E‐4	—	a_*0,P*_ × (1−*C*_T_)	*a*_*1,P*_/*E*_*H*_	—
2 years	0.297	5.316E‐6	7524	*E*_*H*_ × *l*_*2,H*_/*l*_*1,H*_	*a*_*2,P*_/*E*_*H*_	*m*_*2,H*_
3 years	0.116	2.080E‐6	162,337	*E*_*H*_ × *l*_*3,H*_/*l*_*2,H*_	*a*_*3,P*_/*E*_*H*_	*m*_*3,H*_
4 years	0.090	1.618E‐6	384,587	*E*_*H*_ × *l*_*4,H*_/*l*_*3,H*_	*a*_*4,P*_/*E*_*H*_	*m*_*4,H*_

Age/Stage	(c) Fecundity			(d) Both		
	*a*_*x,F*_	*l*_*x,F*_	*m*_*x,F*_	*a*_*x,B*_	*l*_*x,B*_	*m*_*x,B*_
Eggs	*E*_*F*_	—	—	*E*_*F*_	—	—
0 year	*E*_*F*_ × *l*_*0,H*_	*a*_*0,F*_/*E*_*F*_	—	*E*_*F*_ × *l*_*0,H*_	*a*_*0,B*_/*E*_*F*_	—
1 year	*E*_*F*_ × *l*_*1,H*_	*a*_*1,F*_/*E*_*F*_	—	*a*_*0,B*_ × (1−*C*_T_)	*a*_*1,B*_/*E*_*F*_	—
2 years	*E*_*F*_ × *l*_*2,H*_	*a*_*2,F*_/*E*_*F*_	*m*_*2,F*_	*E*_*F*_ × *l*_*2,H*_/*l*_*1,H*_	*a*_*2,B*_/*E*_*F*_	*m*_*2,F*_
3 years	*E*_*F*_ × *l*_*3,H*_	*a*_*3,F*_/*E*_*F*_	*m*_*3,F*_	*E*_*F*_ × *l*_*3,H*_/*l*_*2,H*_	*a*_*3,B*_/*E*_*F*_	*m*_*3,F*_
4 years	*E*_*F*_ × *l*_*4,H*_	*a*_*4,F*_/*E*_*F*_	*m*_*4,F*_	*E*_*F*_ × *l*_*4,H*_/*l*_*3,H*_	*a*_*4,B*_/*E*_*F*_	*m*_*4,F*_

I next used the relationship provided by Audet et al. (2008) given above in equation (1) to convert AW to expected fecundity (*m*_*x,H*_) for each year class (*x*) of mature crabs, where the subscript *H* indicates that this is the historical relationship before the arrival of the Asian shore crab. This size–fecundity relationship was derived from crabs on this same coast, but further north in Canada (as this is the only size–fecundity relationship available for this species), and therefore makes the assumption that this same relationship applies in this study region.

I used the average density of individuals in each year class (*a*_*x*_) as measured in 310 sampling quadrats over a 5‐year period from 1997 to 2001 (Tyrrell 2002). I modeled a 1 m^2^ area and assumed spatial homogeneity throughout the invaded area so that patterns at this small scale can be scaled up to larger spatial scales. Survival of each age class (*l*_*x*_) was calculated from the observed density and the number of eggs as shown in Table 1a. I determined the size‐dependent fecundity in each year class (*m*_*x,H*_) using equation (1) and using the maximum size of crabs in that year class. I then calculated the total number of eggs produced over the modeled area (*E*_*H*_) by combining the size‐dependent fecundity and the density of crabs in each year class (*a*_*x*_):

(3) $$E_{H}=\sum_{x=2-4}m_{x,H}\times a_{x}.$$

I then assessed the sensitivity of population growth rates to predation by the Asian shore crab, to loss of fecundity resulting from interactions with the Asian shore crab, and to the combination of predation and loss of fecundity. I did this by constructing three additional life tables as follows.

The predation impact life table was constructed based on evidence that Asian shore crab predation and cannibalism reduce survival of newly settle green crabs by similar amounts (Lohrer and Whitlatch 2002) and assumed that predation by Asian shore crabs does not appreciably influence the survival of larger green crabs (Table 1b). Thus, I calculated the historic differences in the density of zero‐year class and 1‐year class crabs and assumed that this was due to cannibalism (81.9% mortality). I then assumed that predation by Asian shore crabs would cause the same amount of mortality. However, because an individual green crab that is consumed by a cannibal cannot also be consumed by an Asian shore crab, I combined the impacts of these two predators using the multiplicative risk model that is commonly used to combine the impacts of multiple predators on shared prey (Sih et al. 1998):

(4) $$C_{T}=C_{C}+C_{A}-C_{C}\times C_{A}.$$

where *C*_T_ is total proportional consumption, and *C*_C_ and *C*_A_ are the proportional consumption from cannibalism and Asian shore crabs, respectively. This yielded a combined mortality of 96.7% of zero‐year class green crabs from cannibalism and predation. I assumed that survival and fecundity at all other size classes would be identical to historical levels. I therefore calculated the projected number of crabs in each size class (*a*_*x,P*_) based on observed historical numbers and proportional survival from one size class to the next.

The reduced fecundity impact life table was constructed by assuming that interactions with the Asian shore crab reduce the fecundity of the green crab, but that survival is identical to historical levels for each size class (Table 1c). To do this, I replaced the historical size–fecundity (*m*_*x,H*_) relationship given in equation (4) above, with the size–fecundity relationship reported in this study (see Results for equation). Thus, the total number of eggs produced m^−2^ was determined as:

(5) $$E_{F}=\sum_{x=2-4}m_{x,F}\times a_{x}.$$

The last life table included both the impacts of predation from Asian shore crabs and reduced fecundity to examine their combined impacts (Table 1d). This was made using a combination of the methods described in the two preceding paragraphs. Finally, I calculated the per capita population growth (*R*_0_) for green crabs using each of the four life tables as:

(6) $$R_{0}=\sum l_{x}m_{x}.$$

I then used these population growth rates to project the population forward from a pre‐Asian shore crab density of 79 individuals m^−2^ (Tyrrell 2002) for 5 years postinvasion to examine the relative impacts of these two stressor mechanisms.

## Results

### Field population sampling

Across both sites combined, I collected a total of 171 mature‐sized female crabs. Of these, 46 were gravid, 66 were at various stages of vitellogenesis, and 59 were nonreproductive.

### Size versus diet

**Figure 2:**
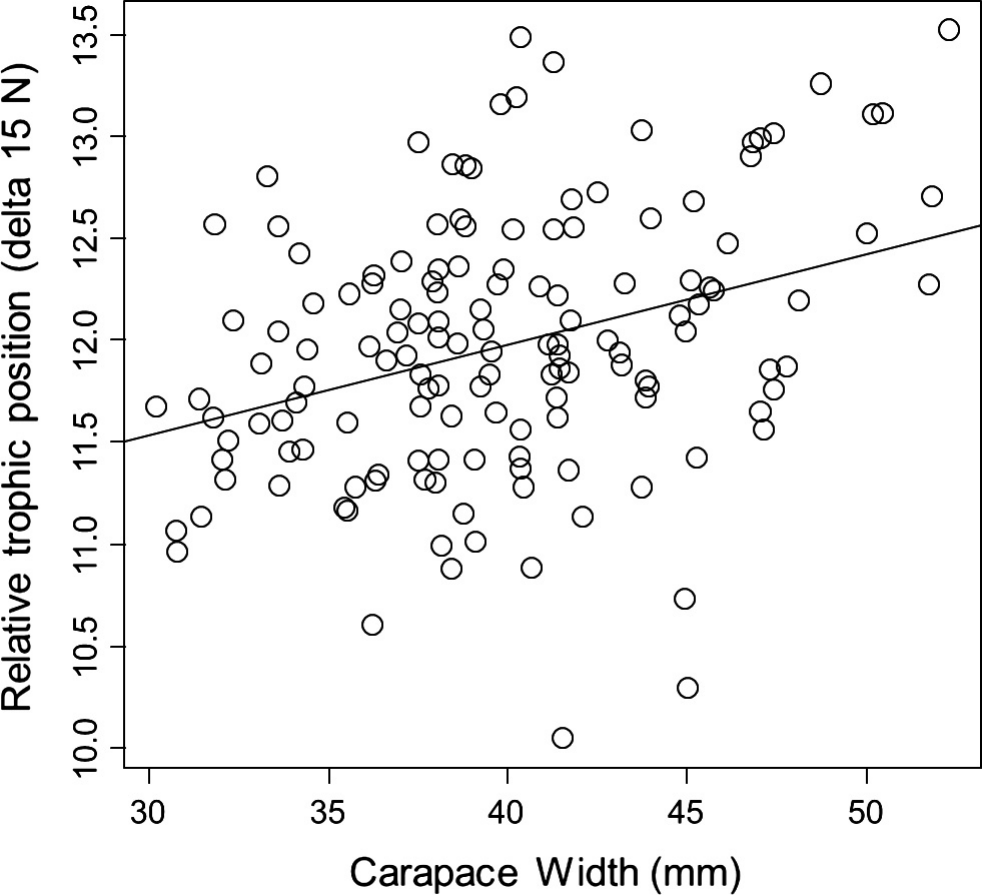
Relative trophic position, as determined using *δ* ^15^N, as a function of carapace width of the green crab *Carcinus maenas* collected from the New Hampshire coast.

I found that for adult female crabs, the relative trophic position, as indicated by *δ* ^15^N, increased with crab size. Overall, relative trophic position increased by approximately 0.044 trophic position with each mm increase in CW (*P* < 0.0001, Fig. 2). However, there was considerable variation in individual trophic position that was not explained by body size (*R* ^2^ = 0.109).

### Size versus fecundity

**Figure 3:**
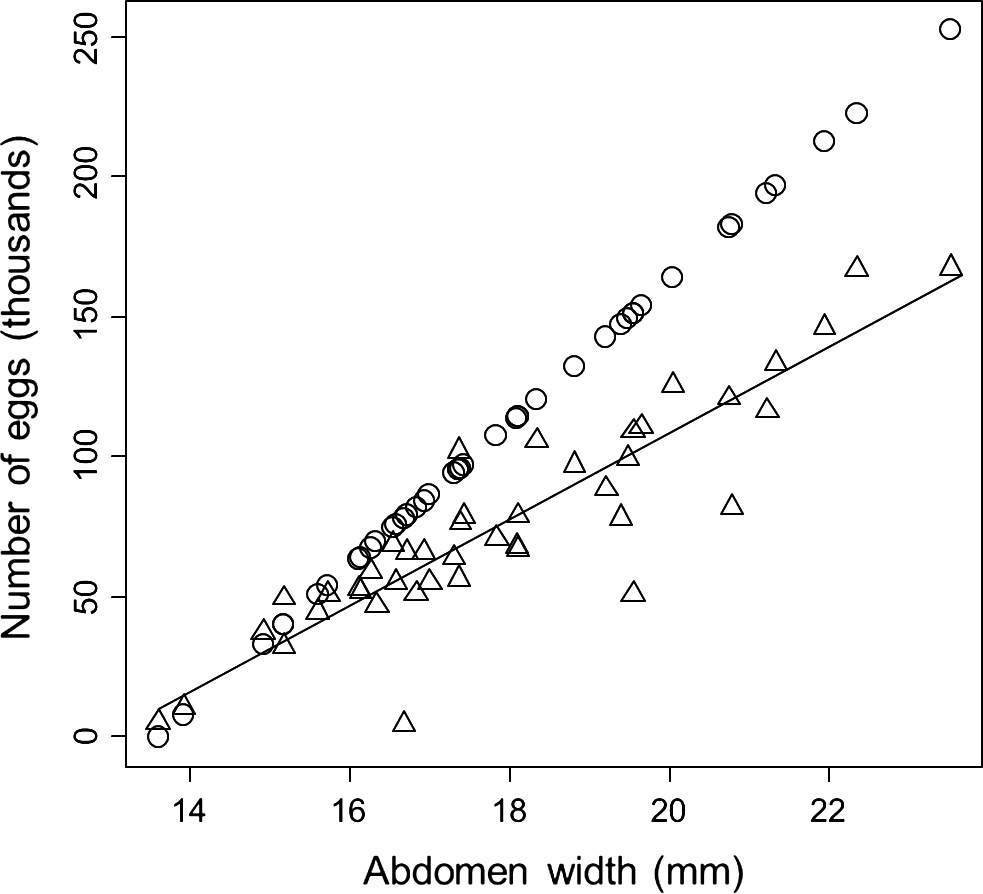
Relationship between the number of eggs being carried versus abdomen width for *Carcinus maenas*. Circles indicate predicted egg numbers based on the relationship given in equation (1) for green crabs further north where *Hemigrapsus sanguineus* are not found. Triangles indicate the observed egg numbers from *C. maenas* sampled on the New Hampshire coast. Regression line is for observed relationship.

As expected, I found that egg production increased strongly with crab size, although it was lower than that predicted using the previously published size–fecundity relationship (*t* = 13.09, *P *< 0.0001, Fig. 3). Overall, the observed clutch size increased by 12,612 eggs with each mm increase in AW (*P* < 0.00001, *R* ^2^ = 0.506, Fig. 3).

### Scaling from individual to population impacts

**Figure 4:**
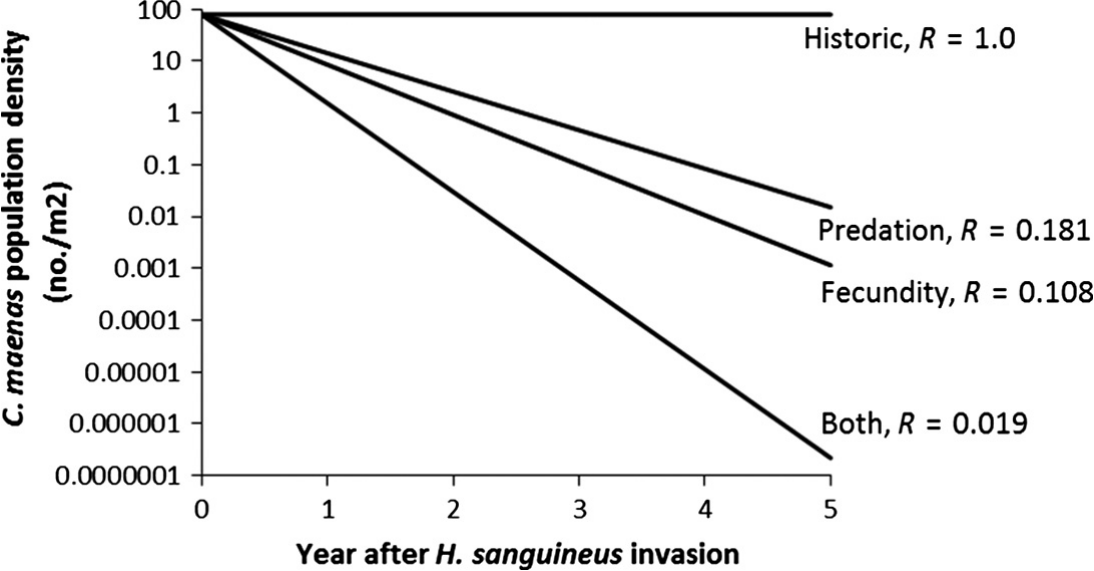
Projected population densities of *Carcinus maenas* in the absence of interactions with *Hemigrapsus sanguineus* (Historic), as a result of predation by *H. sanguineus* on *C. maenas* 0‐year class (Predation), as a result of reduced fecundity caused by diet shifts induced by *H. sanguineus* (Fecundity), and as a result of both predation and reduced fecundity (Both). Projections were calculated using the life tables given in Table 1.

Based on the mean of the densities of green crabs at Odiorne Point State Park from the 5 years preceding the invasion of this site by the Asian shore crab, the historic per capita population growth rate (*R*_*0*_) of green crabs at this site was 1.0, indicating a stable population (Table 1a). Assuming that cannibalism and predation can have similar impacts on green crab survival, and combining these two sources of mortality using the multiplicative risk model, resulted in predation by Asian crabs alone reducing the per capita population growth rate of green crabs to *R*_*0*_ = 0.181. Replacing the historic fecundity levels with the size–fecundity relationship reported here, reduced fecundity of green crabs because of interactions with Asian shore crabs decreases the per capita population growth rate to *R*_*0*_ = 0.108. And combining the impacts of predation and reduced fecundity simultaneously further reduces the green crab per capita population growth rate to *R*_*0*_ = 0.019. When these respective per capita population growth rates were used to project the population forward from its historic mean density of 79 individuals m^−2^, it was evident that both predation and loss of fecundity are capable of drastically reducing green crab population levels over short time scales that are consistent with previously documented declines of this species. Overall, this analysis suggests that loss of fecundity was slightly more detrimental than predation, although both were capable of producing the previously observed declines in green crab populations with the arrival of the Asian shore crab (Fig. 4).

## Discussion

I have shown that diet of adult European green crabs is highly variable and that while animal consumption does increase with body size, there is considerable individual diet variation not explained by body size. This residual variation may reflect spatial variation in food availability, individual dietary specialization (Bolnick et al. 2003), and/or variation in the intensity with which interactions with the Asian shore crab influence individuals of this species. In addition, I have also shown a familiar size–fecundity relationship for green crabs (Audet et al. 2008). However, variation in the relationship seen here was much greater than that seen in this species previously (*R* ^2^ = 0.506 in this study vs. *R* ^2^ = 0.916 previously reported in Audet et al. 2008). This may be a function of the large variation in trophic position observed here, because animal consumption, but not plant consumption, increases fecundity in green crabs (Griffen 2014).

Not only was there greater variation in the size–fecundity relationship than has previously been seen for this species, I also found much lower individual fecundity overall than has previously been reported for this species (Broekhuysen 1936; Crothers 1967; Audet et al. 2008). In fact, only the three largest crabs sampled had fecundity anywhere in the range previously reported for this species (140,000–200,000 eggs). I based this study on the assumption that previously demonstrated impacts of Asian shore crabs on green crab diet and reproduction in this same area (Griffen et al. 2008, 2011) are responsible for this decline in fecundity. However, I did not directly measure the impacts of Asian shore crabs on fecundity of the individual green crabs used in this study, and it is possible that factors other than interactions between these species were responsible for some of the observed trends. For instance, prey recruitment at this site is temporally variable (Griffen and Byers 2009), and poor prey recruitment could limit food availability and would therefore contribute to the patterns seen here. It is not known whether this occurred.

The life‐table analysis used here to scale up to population impacts included several assumptions. For instance, it assumed that all females were reproductive, which was, in fact, not the case. I found that only 65% of crabs were gravid or vitellogenic at the time of capture. This is similar to previous studies in this region that found approximately 75% of captured females to be gravid (Ropes 1968), although the number that may have been vitellogenic in this previous study is unknown. Nonreproductive females could be incorporated into the life‐history analysis by modifying the survival function. However, I did not do this because individuals that did not reproduce this year may reproduce next year, and it is uncertain whether a constant proportion of the female population reproduces each year, or how or whether this may vary for different size classes.

The life‐table analysis here also assumed that the size–fecundity relationship observed by Audet et al. (2008) in Canada, shown in equation (1), is also valid at my study sites on the New Hampshire coast. This assumption only influences the calculation of *R*_*0*_ from the historic life table. This assumption may not be strictly correct, as fecundity is known to vary with latitude for other crustaceans (Wägele 1987). However, this relationship yielded a per capita population growth rate *R*_*0*_ = 1.0, indicating a stable population that was neither growing nor shrinking. This is consistent with green crab populations that were stable in this area for many decades before the arrival of Asian shore crabs. Thus, this size–fecundity relationship measured further north may not be too dissimilar from the historic size–fecundity relationship at my sites.

Finally, the life‐history analysis used here assumed spatial homogeneity throughout the invaded area, allowing direct inference from the 1 m^2^ over which calculations were conducted to larger spatial scales (i.e., to populations). This assumption is likely valid, or nearly so, over relatively small spatial scales, such as within a single beach. However, over larger spatial scales, there is spatial heterogeneity that offers green crabs a spatial refuge. For instance, green crabs are common in both salt marsh (Hampel et al. 2003) and subtidal habitats (Hunter and Naylor 1993). And while Asian shore crabs have been found occupying fiddler crab burrows in salt marsh habitats (Brousseau et al. 2003) and inhabiting subtidal habitats (Gilman and Grace 2009), they are not abundant in either of these habitats and likely interact with green crabs in these habitats only weakly. Spillover from these refuge habitats may explain the persistence of green crabs at low abundance in rocky habitats where Asian shore crabs thrive. Lastly, there is also a latitudinal gradient, with Asian shore crabs being more abundant toward Long Island Sound than in more northern regions of the Gulf of Maine. Thus, the predation and reproductive impacts of Asian shore crabs on green crabs are likely to also be latitudinally dependent.

### Scaling from individuals to population responses to stressors

I used life tables to scale up multiple pathways of interaction between two invasive species. My analysis showed that both predation and reduced fecundity are capable of producing the declines in green crab abundance that have been observed with the introduction of the Asian shore crab. The predominant mechanism responsible for the observed dynamics may potentially vary spatially and/or temporally, but the strength of both mechanisms may be expected to vary together. For instance, predation and lost fecundity may both be expected to decrease in importance in years with high recruitment of mussels, barnacles, or other important prey species. High abundance of important prey species may reduce predation on green crabs by providing Asian shore crabs with alternative diet choices and may reduce losses of fecundity by reducing exploitative food competition between the two species. Additionally, the predominance of intertidal habitat structure (i.e., boulders) and the reduced density of Asian shore crabs should mean that interactions between these species are less intense in northern regions (e.g., Maine) as compared to southern regions (e.g., Long Island Sound), and therefore, both predation and lost fecundity should decrease with latitude.

Given increasing human populations and attending human‐induced stressors in natural systems, accumulating stressors mean that many systems are dealing with multiple stressors simultaneously. As a result, the combined impacts of multiple stressors have become an important area of research (e.g., reviewed in Heugens et al. 2001, Crain et al. 2008). The life‐table approach used here provides a viable method for examining the population‐level impacts of multiple stressors, as long as the individual mechanistic response to the stressor can be linked back to impacts on survival or fecundity.

## Conflict of Interest

None declared.
